# Bridging the gap between laboratory and applied research on response‐independent schedules

**DOI:** 10.1002/jaba.965

**Published:** 2022-11-28

**Authors:** Einar T. Ingvarsson, Eduardo J. Fernandez

**Affiliations:** ^1^ Virginia Institute of Autism; ^2^ School of Education and Human Development University of Virginia; ^3^ School of Animal and Veterinary Science University of Adelaide

**Keywords:** adventitious reinforcement, induction, noncontingent reinforcement, response‐independent schedules, superstition

## Abstract

In 1948, Skinner described the behavior of pigeons under response‐independent schedules as “superstitious,” and proposed that the responses were reinforced by contiguous, adventitious food deliveries. Subsequently, response‐independent schedules have been of interest to both basic and applied researchers, first to understand the mechanisms involved, and later, as “noncontingent reinforcement” (NCR) to reduce undesirable behavior. However, the potential superstitious effects produced by these schedules have been challenged, with some researchers arguing that antecedent variables play a significant role. This paper examines the evidence for adventitious reinforcement from both laboratory and applied research, the results of which suggest that antecedent, nonoperant functions may be important in fully understanding the effects of NCR. We propose an applied‐basic research synthesis, in which attention to potential nonoperant functions could provide a more complete understanding of response‐independent schedules. We conclude with a summary of the applied implications of the nonoperant functions of NCR schedules.

In the science of behavior analysis, response‐independent schedules involve the delivery of stimuli based on the passage of time (e.g., fixed or variable‐time schedules). The stimuli are typically either items or events that have been shown to function as reinforcers in previous assessments (functional analyses or reinforcer assessments), or stimuli that are presumed to function as reinforcers due to presession manipulations (such as when food is withheld from an organism for a period of time). Thus, response‐independent schedules constitute an experimental preparation in which a positive contingency between the response and the “reinforcer” is absent (Borrero et al., 2002).^1^ Practitioners and researchers in applied behavior analysis routinely use response‐independent schedules (often called “noncontingent reinforcement,” or NCR) either as reinforcement control procedures (Thompson & Iwata, 2005) or as behavior‐reduction procedures (Richman et al., 2015). Throughout the last few decades, applied research on NCR and basic research on response‐independent schedules has accumulated, but the lines of research appear to have remained largely independent. Specifically, it is not clear that relevant basic research findings have influenced applied research or practice on NCR, nor is it apparent that the tools of basic research have been brought to bear on questions relevant to application.

Much of the basic research on response‐independent schedules was initially influenced by B. F. Skinner's paper, “‘Superstition’ in the Pigeon” (1948), and the concerns raised by this paper continue to resonate when the potential limitations of NCR schedules are discussed. Skinner's paper has been widely cited both within and outside of the field of behavior analysis as providing an explanation of superstitious and “irrational” behavior, and by extension, demonstrating the power of consequences to influence behavior in general. In Skinner's study, response‐independent (i.e., time‐based) delivery of food reinforcement ostensibly resulted in the development of varied “superstitious” responses, which differed between pigeons. This finding appears to be frequently accepted with few reservations, particularly outside of the relatively limited confines of basic behavioral research. As an example, Cooper et al. (2020), in their widely assigned and authoritative text *Applied Behavior Analysis*, cite the study as a powerful demonstration of how adventitious (or “accidental”) reinforcement can strengthen idiosyncratic or arbitrary behavior (while cautioning against the notion that all superstitious behavior comes about through such history). Outside of behavior analysis, popular science media sometimes cite Skinner's finding as an accepted explanation of superstitious behavior (e.g., Inglis‐Arkell, 2011; Lallania, 2013).

Skinner's superstition research has cast a considerable shadow over subsequent research and application, with concerns being raised that clinical application of NCR schedules could result in adventitious reinforcement and maintenance of *idiosyncratic* responses (i.e., responses that are unique to the individual) that would otherwise not have occurred. A related, but distinct matter involves transitions from response‐contingent to response‐independent schedules, most commonly seen when function‐based NCR is implemented following functional analyses. A commonly raised concern is that the target response (usually undesirable behavior) could be maintained through accidental response–reinforcer contiguities under such arrangements (Newman et al., 2021; Smith, 2021). An additional concern has to do with the occurrence of behavior maintained by a change between schedules, particularly those of similar response topographies, or compatible concurrent operants (Catania, 1966; Catania & Cutts, 1963).

Yet, subsequent research has cast doubt on Skinner's findings, suggesting that response‐independent delivery of reinforcement is unlikely to result in idiosyncratic responses, but may instead elicit or induce respondently conditioned or species‐typical response patterns related to *foraging* (searching for and obtaining food and provisions; e.g., Fernandez & Timberlake; 2020; Staddon & Simmelhag, 1971; Timberlake & Lucas, 1985). Similarly, the evidence for maintenance of previously reinforced responses under NCR schedules is limited, and such effects seem to be rare and fleeting (e.g., Rey et al., 2020a; 2020b; Zeiler, 1968). If Skinner's original findings are correct, superstitious behavior and maintenance of previously reinforced behavior should be a common outcome of these procedures. Yet, practitioners in applied behavior analysis continue to implement NCR procedures, seemingly without much worry that such procedures might lead to idiosyncratic superstitious behavior or maintenance of problem behavior for their students and clients (e.g., Phillips et al., 2017).

In this paper, we provide a general overview of the idea that response‐independent schedules generate superstitious responding or other kinds of adventitious reinforcement or maintenance of undesirable behavior and discuss alternative theoretical accounts that have gained traction in the basic literature since Skinner's original idea. It seems probable that much of the behavior that has been described as superstitious is not operant at all but may instead be either species‐specific or otherwise evoked by antecedent events (Killeen, 1978; Staddon, 1992; Timberlake, 2004 **)**. Along similar lines, we discuss the possibility that competing behavior, evoked by antecedent events, may explain at least some of the suppressive effects of NCR schedules, which make them useful as interventions for undesirable behavior and as control conditions for the effects of operant reinforcement schedules. We then outline avenues of research that might help further elucidate the multiple effects engendered by response‐independent schedules and discuss the potential implications for the use of NCR in applied behavior analysis.

## Basic Research on Superstition and Adventitious Reinforcement

As noted above, Skinner (1948) described the responses of pigeons produced by response‐independent schedules under which food is delivered at fixed times, independent of behavior, as *superstitious*. Skinner argued that responses occurring immediately prior to the delivery of food were reinforced and therefore more likely to occur (and be rewarded) in the future. In support of his interpretation, Skinner described several idiosyncratic response patterns that emerged for individual pigeons. Some of these behaviors included turning counterclockwise about the cage, tossing the head by dropping it and lifting it repeatedly, and side‐to‐side stepping or hopping. Skinner likened the behavior of pigeons under these fixed‐time (FT) schedules to the superstitious behavior of humans and proposed that both response patterns were the result of contiguous pairings of rewards following some response.

### The Superstition Argument

Skinner (1948) used several arguments based on his observations to support the claim of contiguous, adventitious reinforcement resulting in superstitious behavior. One claim, as noted above, was that the responses were idiosyncratic across almost all the pigeons, thereby suggesting that the behaviors were arbitrary and not simply a result of species‐typical response patterns. Another claim suggested that one bird that produced a stepping/hopping response pattern experienced an extinction schedule until few of the stepping/hopping responses occurred within a 10–15‐min interval. When the bird was once again exposed to the response‐independent food schedule, a new behavior was reported to emerge, which consisted of walking around the cage. The previous behavior of stepping/hopping from side‐to‐side was stated to have never reappeared. Finally, Skinner compared the behavior of the pigeons under response‐independent schedules to that of humans engaging in superstitious or ritualistic behaviors and suggested that both were the result of accidental response–reward contingencies. It is worth noting that in almost all the examples noted above, Skinner's descriptions were based on anecdotal observations. Skinner's original superstition paper only presented one instance of quantitative data, and that was of a cumulative record of steps counted via a tambourine‐like device and in the absence of any clearly discernable response topography.

Since Skinner's (1948) description of superstitious behavior, adventitious reinforcement has been used to explain several other phenomena, including the maintenance of avoidance rituals in agoraphobics (Thyer, 1986), compulsions and obsessions (Herrnstein, 1966), visceral conditioning in biofeedback procedures (Linton, 1978), fear of nausea and vomiting (Klonoff et al., 1984), and the maintenance of behaviors in artificial neural networks (Burgos, 2000). However, the extent to which evidence exists for adventitious reinforcement is open to question. The following sections examine the evidence for the maintenance of target responses under response‐independent schedules and reinforcement of nontargeted, idiosyncratic behavior.

### Superstition: Pigeons and Rats

Rather than studying the potential effects of response‐independent schedules on idiosyncratic response topographies, much of the initial research that followed Skinner's (1948) original findings focused on transitions from contingent (response‐dependent) to response‐independent schedules. This was accomplished by initially training pigeons to peck a key on a fixed‐ or variable‐interval schedule (FI and VI, respectively) where some amount of time must occur before a response is reinforced. After training the pigeons to maintain key‐pecks on the FI or VI schedules, response‐independent schedules were presented, which included intervals of similar durations to the previous FI or VI schedules (Appel & Hiss, 1962; Herrnstein & Morse, 1957; Lachter et al., 1971; Morse & Skinner, 1957; Neuringer, 1970; Zeiler, 1968). According to an adventitious reinforcement explanation of superstition, key‐pecking should be maintained under the response‐independent schedules because they were produced immediately prior to food deliveries on the response‐dependent interval schedules (Fernandez & Timberlake, 2020; Timberlake & Lucas, 1985). However, in many of the above cases, key‐pecks decreased in frequency during the response‐independent schedules, often to near zero rates of responding. Although it may be argued that ‘something else’ may have been contiguously reinforced, this cannot explain all reductions in key‐pecking, particularly when various durations (i.e., 5 – 240 s) are trained under response‐dependent (FI) schedules and then matched to the same response‐independent (FT) times (Lachter et al., 1971). Thus, contiguity alone cannot maintain any form of key‐pecking.

In several of these studies, incidental stimuli in the form of different colored lights imposed on the key or flashing overhead lights were used during the FI and VI schedules and were then associated with some rate of key‐pecking during the response‐independent schedules that followed (Herrnstein & Morse, 1957; Morse & Skinner, 1957; Neuringer, 1970). However, Brown and Jenkins (1968) demonstrated that the use of incidental stimuli in the form of lighted keys prior to the delivery of food could elicit key‐pecking as a result of respondent conditioning. This procedure, described as *autoshaping*, has been shown to effectively maintain key‐pecking, even when an omission contingency that should otherwise punish such responding is introduced (Williams & Williams, 1969). Without proper experimental control for the administration of these incidental visual stimuli delivered prior to food deliveries, it is unclear if the key‐pecking observed in the above studies was the result of adventitious reinforcement or if it was elicited as the result of light–food respondent pairings.

#### Evidence for Idiosyncratic Behavior

Later superstition studies attempted to measure idiosyncratic response topographies, either with rats (Davis & Hubbard, 1972; Reberg et al., 1978; Rescorla & Skucy, 1969) or pigeons (Eldridge et al., 1988; Justice & Looney, 1990). In some cases, responses were not clearly defined, and lack of responding on one topography, or even the occurrence of only one response topography, was used as proof for or against adventitious reinforcement as an adequate explanation of superstitious behavior. However, Skinner's (1948) initial paper on superstition focused on direct observation of multiple response topographies and idiosyncratic descriptions of the observed response patterns. Anecdotal as those observations may have been, it illustrated the need for systematic measurement of behaviors beyond one mechanically recorded response topography to properly study the effects of response‐independent schedules (Davis et al., 1973; Fernandez, 2021). In other words, to properly study the potential idiosyncratic effects of adventitious reinforcement, one needs to systematically measure a variety of possible responses.

Two such studies on response‐independent schedules and superstition involving pigeons quantified multiple response topographies, with both offering alternative explanations to adventitious reinforcement: Staddon and Simmelhag (1971) and Timberlake and Lucas (1985). Staddon and Simmelhag coded the behavior of pigeons over multiple days of FT presentations and described two predominant classes of responding: interim and terminal behaviors. Interim behaviors, such as circling, pecking at the floor, and moving along the front panel, peaked in the middle of the interfood interval before food was made available. Terminal behaviors, such as orienting toward the food hopper and pecking in and around it, were observed primarily at the end of the interfood interval, just before food appeared. Staddon and Simmelhag argued that the behaviors displayed by the pigeons were more like adjunctive behaviors and, thus, schedule‐induced rather than directly controlled by adventitious reinforcement. They also concluded that the preponderance of pecking by their animals was related to the respondent process of stimulus/response substitution in which the temporal CS substituted for the food (the US) in eliciting pecking (the UR).

Timberlake and Lucas (1985) also coded the behavior of pigeons in nine experiments and found results more consistent with the account of Staddon and Simmelhag (1971) than Skinner (1948). They reported that nearly all the birds turned and circled away from the hopper following the delivery of food, returning to the hopper area partway through the interval. However, instead of the response of pecking emphasized by Staddon and Simmelhag, they found stepping, head bobbing, and pressing the breast against the wall around the hopper. The surprising similarity of the behaviors that emerged for individual pigeons cast considerable doubt on the adventitious reinforcement/superstition explanation of Skinner. Further questions of the adventitious reinforcement account were raised by experiments in which pigeons were trained briefly either to peck or turn on FI 15‐s schedules and then successively introduced to FT 15‐s schedules, or placed on an omission contingency (i.e., response cost condition) from the beginning of acquisition that withheld food deliveries if the bird was near the hopper during the last 3 s of the schedule. In both cases, the pigeons tended to show similar wall‐directed behavior.

The theoretical account offered by Timberlake and Lucas (1985) was that the behavior that emerged under FT schedules represented a compressed form of naturally occurring food seeking (foraging) bouts on the part of the pigeons with most of the components that occurred afforded by the schedule of food delivery, as well as the delivery of food itself (see also Timberlake, 1997; Timberlake & Lucas, 1989; Timberlake & Silva, 1995). For example, the turning and circling behaviors resembled the behavior of pigeons searching in a field for seeds and grain. Other researchers demonstrated that ring‐necked doves, a closely related species to pigeons, engaged in similar nonidiosyncratic patterns of responding under response‐independent schedules of food delivery, but that chickens under the same schedules engaged almost exclusively in scratching and floor pecking behaviors, both of which are species‐typical foraging patterns for their wild counterparts (Fernandez & Timberlake, 2020).

### Superstition: Humans

Several studies have experimentally examined response‐independent schedules and superstitious behavior for language‐capable humans (Aeschleman et al., 2003; Benvenuti et al., 2018; Bloom et al., 2007; Catania & Cutts, 1963; Heltzer & Vyse, 1994; Mellon, 2009; Ninness & Ninness, 1998; Ono, 1987; Rudski, 2000; Rudski et al., 1999; Wagner & Morris, 1987). Many of these studies encounter difficulties in interpretation, including (a) assumptions that any increase in behavior is a result of an operant contingency, as opposed to being elicited or schedule‐induced, (b) a lack of idiosyncratic responding for many of the subjects, and (c) reliance on the subjects' self‐reports of causal inference, as opposed to direct performance during the experimental task. Several of these studies are discussed below.

Ono (1987) examined superstitious conditioning with 20 undergraduate students who were given three possible levers to operate on either a FT or VT (variable time) 30 or 60‐s response‐independent schedule. Of the 20 students who participated, only three were reported to develop consistent superstitious behaviors. Therefore, even if superstitious conditioning occurred due to the experimental contingencies, it does not appear to be very common.

Aeschleman et al. (2003) instructed subjects to either keep the word “good” on a computer screen (positive reinforcement group), or to stop the word “bad” from appearing on the computer screen (negative reinforcement group). The words appeared on the screen independent of responding, either every 6 s or 6 min (Experiment I), or every 15 s or never (Experiment II). Subjects were then asked to rate their ability to control the word that appeared on the screen and their confidence in discovering a method that controlled the word appropriately. For both experiments, the subjects in the leaner negative reinforcement group (6 min and never) reported a significantly greater confidence in controlling the word compared to the other groups. In addition, in Experiment I, the subjects in the negative reinforcement 6‐min group reported significantly greater control over the stimulus. The authors interpreted their data as suggesting that superstition is more likely to occur when subjects are adventitiously rewarded for escaping aversive stimuli, and that this is also more likely to occur when the aversive stimuli are infrequent or do not occur at all.

Aside from the challenges in assessing all of the possible controlling variables in these studies, there remains the problem with studying the effects of response‐independent schedules with language‐capable subjects who receive instructions on some aspect of their performance. Although Aeschleman et al. (2003) reported that response‐independent schedules produced superstitious behavior, this was based on the vocal‐verbal reports of the subjects in controlling the contingencies, not their actual performance. This is a confound for any explanation of superstitious behavior by language‐capable subjects occurring due to adventitious reinforcement: Are the causal variables relevant to the behavior observed a result of the response‐independent schedules, or is the cause–effect verbal behavior (covert or overt), generated by the participants? It seems at least equally plausible that a long history of responding in accordance with rules as contingency‐specifying stimuli may result in verbally capable participants generating their own rules to account for occasional coincidental behavior–consequence pairings (Blakely & Schlinger, 1987; Schlinger & Blakely, 1987). Verbally mediated superstitious behavior is certainly an important and interesting topic of study in its own right, but research on superstitious human behavior does not appear to provide convincing evidence of adventitious reinforcement of either idiosyncratic or target behavior.

#### Concurrent Superstition

Catania and Cutts (1963) examined the possibility of concurrent superstition (Catania, 1966) where presses on one button were reinforced on a VI 30‐s schedule, whereas presses on another button were never reinforced. In their study, most of the subjects responded on both buttons, even though responses on only one of the buttons were reinforced contingently. Responses on the nonreinforced button were then eliminated when a changeover delay (COD) was implemented. The main difficulty with describing such responding as “superstitious” is that it requires presuming that the increased responses observed on the non‐directly‐reinforced button were the result of reinforcement. However, similar to the second type of superstition proposed by Morse and Skinner (1957), the effects observed may be the result of the implementation of the contingency changes itself, which could include antecedent effects. As we discuss throughout this review, antecedent effects may account for many of the changes in behavior that are often presumed to be operant. In any event, these results suggest that when one topography is placed on a schedule of contingent reinforcement, other similar topographies might occur and be maintained in the absence of an explicit contingency.

### Basic Research on Response‐Independent Schedules Summarized

The concept of adventitious reinforcement and superstition provided a useful heuristic for the promotion of operant conditioning principles. Nevertheless, several difficulties exist in attempting to use adventitious reinforcement as an explanation for the occurrence of behavior under response‐independent schedules. First, pigeons previously trained to peck a key under response‐dependent (i.e., fixed‐ or variable‐interval) schedules generally do not continue to do so when exposed to similar response‐independent schedules. Second, when multiple behaviors are directly measured under response‐independent schedules, the topographies of those responses are not idiosyncratic. The production of idiosyncratic responses under response‐independent schedules is an essential argument for the demonstration of adventitious reinforcement because the lack of varying topographies suggests that the behaviors observed are not arbitrary and, therefore, are a result of events other than contiguous response–reward associations. Third, the existing laboratory human research on response‐independent schedules and superstition suffers from both a lack of consistently observed effects and the potential confounds inherent in a subject's verbal or otherwise rule‐governed behavior.

In short, the behavior observed in the laboratory under response‐independent schedules does not appear to support an adventitious reinforcement interpretation. Instead, the behaviors that do emerge appear to be more causally related to species‐typical responding afforded by the type and frequency of stimuli delivered, as well as the species observed. Skinner readily acknowledged the importance of natural selection and an organism's species‐typical behavior in relation to what is and can be learned (Skinner, 1981; 1987). The behavior of organisms under response‐independent schedules should likewise be understood within such a framework, with consideration for the potential nonoperant functions that almost certainly play an important role.

## Response‐Independent Schedules and Applied Behavior Analysis: Noncontingent Reinforcement (NCR)

In the research and practice of applied behavior analysis, response‐independent schedules are commonly referred to as NCR (noncontingent reinforcement). NCR can be viewed as a procedure (or, a set of procedures) involving the delivery of stimuli, previously demonstrated to function as reinforcers for some behavior, in a manner that is not contingent on a particular target response. This can take the form of FT stimulus delivery (Vollmer et al., 1993), VT stimulus delivery (Van Camp et al., 2000), continuous stimulus delivery (Hernandez et al., 2007), or even trial‐based stimulus delivery (Ingvarsson et al., 2009). Further, NCR can take the form of removal or withholding of stimuli, as in the case of the delivery of noncontingent breaks in the treatment of escape‐maintained problem behavior (Vollmer et al., 1995). NCR is generally used for one of two purposes in applied and translational research: as a reinforcement control procedure (Thompson & Iwata, 2005), and as an intervention for aberrant behavior (Carr et al., 2000).

### Examples of NCR as a Reinforcement Control Procedure

Hanley et al. (2014) found that challenging behavior occurred reliably when synthesized reinforcers (e.g., escape from task demands and access to preferred items) were delivered contingently, but reduced to near zero levels when the same reinforcers were delivered noncontingently and continuously. In other studies, lower rates of target responding during NCR control conditions led the researchers to conclude that increases in target behavior were likely due to the contingent delivery of the stimuli in question, rather than their mere presence (Rescorla & Skucy, 1969; Thompson & Iwata, 2005). In a different line of research, NCR (reported as response‐independent schedules) has also been used as a control to study the effects of food alone during shaping (Fernandez, 2020; Fernandez & Rosales‐Ruiz, 2020).

However, NCR control conditions do not always result in the expected reduction in behavior (e.g., Thompson et al., 2003). As an example, in a study of 3‐month‐old infants, Bloom and Esposito (1975) found that noncontingent delivery of social stimulation produced roughly the same rate of infant vocalizations as contingent stimulation. In a second experiment, they found that a 5‐s DRO schedule produced the same rate of vocalizations as response‐independent interaction. Similarly, Masataka (1993), in a study of 2–3‐month‐old infants, found that contingent and noncontingent parental attention increased responding over a no‐attention baseline, but the difference between the two conditions was not statistically significant. The results of these studies show that some level of responding can be maintained in NCR conditions. At issue here is the second concern described above: adventitious maintenance of responding that was previously maintained through an operant contingency. Thompson and Iwata (2005) noted that although adventitious reinforcement has been suggested as a possible explanation for maintenance of behavior under these conditions, there is scant evidence of this occurring. Further, responding sometimes persists even when a negative contingency is arranged, as in omission or DRO contingencies (e.g., Thompson et al., 2003; Williams & Williams, 1969). Persistence of behavior under these conditions does not seem to be completely explained by reference to response‐strengthening (or response‐selecting) effects of operant reinforcement.

### NCR as a Behavior‐Reduction Procedure

In discussing NCR as a treatment procedure, Holden (2005) related the following anecdote:

I attended a paper session on NCR at the ABA convention in 1997, and I remember a comment afterwards: the whole approach was unrealistic! Probably having Skinner's “superstition” experiment in mind, my colleague thought that NCR would inevitably result in reinforcement of problem behavior. Such a reaction is understandable. However, so far research has largely failed to nurture this pessimism. (p. 4)

This pessimistic prediction has indeed failed to materialize, and NCR has been shown to be an effective treatment for problem behavior. In a meta‐analysis of single‐case studies, Richman et al. (2015) found a strong effect size (*d* = ‐1.58) for reductions in problem behavior. Similarly, in a consecutive case study of 27 applications, Phillips et al. (2017) found that NCR was generally effective for socially maintained challenging behavior, whereas additional treatment components were often needed for automatically reinforced behavior. Nevertheless, leading researchers in applied behavior analysis still cite adventitious operant reinforcement (both adventitious maintenance of previously reinforced behavior and adventitious reinforcement of “idiosyncratic behavior) as an important concern. Smith (2021) noted that “NCR may produce adventitious reinforcement of unspecified behavior, which may include target or other problem behavior” (p. 310). Newman et al. (2021) speculated that a “possible explanation for increases in compliance observed during NCR could be due to adventitious reinforcement … edible or escape delivery may have been contiguous with compliance, resulting in a positive contingency…” (p. 997). The adventitious reinforcement hypothesis seems common when unexpected outcomes of NCR occur.

In a typical NCR application, a functional analysis is conducted to elucidate a probable function of the aberrant behavior (Iwata et al., 1982/1994). The functionally relevant reinforcer is then delivered independent of responding (often on a time‐based schedule), typically resulting in a decrease in problem behavior. For example, Vollmer et al. (1993) found that the self‐injurious behavior of three women with developmental disabilities was maintained by contingent delivery of attention. A treatment in which attention was delivered according to a FT schedule—but withheld following self‐injury—was effective in reducing the rate of self‐injury for all participants.

NCR involves the removal of the response–reinforcer contingency (or at least, dependency); that is, the reinforcer is presented independent of the occurrence of the target response. Applied researchers have often looked for operant explanations for reductions in problem behavior under NCR in terms of either operant extinction (e.g., Vollmer et al., 1995) or satiation or other abolishing operations (Laraway et al., 2003). Applied research has revealed patterns of responding consistent with both hypotheses: Some participants do not show increases in responding during an extinction session immediately following an NCR session, suggesting that extinction may have taken place during NCR, whereas others show increases in responding during extinction, suggesting a transition from a state of satiation to a state of deprivation (Kahng et al., 2000).^2^ Although it is plausible that these explanations may account for at least some of the outcomes of NCR schedules, neither of these accounts explains the effects of NCR using non‐function‐based reinforcers. Several applied studies have shown that NCR that involves the delivery of functionally irrelevant (alternative) reinforcers can be equally effective, even when the functionally relevant reinforcer is still delivered contingent on challenging behavior (Ingvarsson et al., 2008; Lomas et al., 2010; Newman et al., 2021; Payne & Dozier, 2013). For example, Lomas et al. (2010) found that the challenging behavior of three boys with autism and other developmental disabilities was maintained by escape from demands. During the treatment analysis, challenging behavior resulted in a 20‐s break from instructions (the functionally relevant reinforcer); additionally, the therapist delivered a highly preferred edible and a brief praise statement on a 15‐s FT schedule (functionally irrelevant/alternative reinforcer). The intervention resulted in reductions in problem behavior and increased compliance for all three participants. In their study of 27 consecutive cases, Phillips et al. (2017) reported that NCR applications involving functionally relevant reinforcers, functionally irrelevant reinforcers, or combination of both were equally effective. Additionally, in their meta‐analysis of the NCR treatment literature, Richman et al. (2015) found that NCR arranged with functionally irrelevant reinforcers was only slightly less effective than NCR using functionally relevant reinforcers; this variable accounted for about 10% of the variance in problem behavior.

Adventitious reinforcement of incompatible or alternative behavior may seem like a possible explanation of the effects of NCR. This interpretation is, at first glance, consistent with results that show that functionally irrelevant reinforcers work almost as well as functionally relevant reinforcers (Ecott & Critchfield, 2004; Virues‐Ortega et al., 2013) and sometimes lead to increases in alternative behavior, such as compliance or cooperation with instructions, in the absence of an explicitly arranged contingency (Ingvarsson et al., 2008). If this outcome were to be demonstrated experimentally, it would presumably be an example of adventitious reinforcement of behavior previously maintained through an operant contingency. However, consistent with the superstition literature reviewed above, no evidence of adventitious reinforcement has been found when researchers have collected data on response–reinforcer contingency (e.g., Ingvarsson et al., 2008; Ingvarsson et al., 2009; Lomas et al., 2010; Virues‐Ortega et al., 2013). Further, the immediate reductions in problem behavior that often occur when NCR (function‐relevant or function‐irrelevant) is implemented are inconsistent with the adventitious reinforcement hypothesis (Lomas et al., 2010).

Similar findings have been reported by Rey and colleagues in their research on DRO schedules with college students (Rey et al., 2020a; 2020b). The authors found that although alternative responses appeared to occasionally contact an adventitious contingency in the VT schedule control condition, as shown by temporary increases in response rates, this outcome was fleeting and unreliable. In Rey et al. (2020a) three of nine participants showed temporarily elevated responding under VT schedules, but these effects were not replicated within participants. Overall, the contingency‐strength value for the alternative response in the VT condition was ‐0.55, indicating negative contingency (i.e., reinforcers were less likely to occur following target behavior than at other times). The findings of Rey et al. (2020b) were similar; in the rare cases in which the within‐session contingency‐strength value was positive (which occurred in one session for four out of nine participants), the effect was not replicated within participants. However, as with other studies that have included measurement of other behavior (e.g., compliance with demands), the measurement was limited to a restricted number of topographies, and it is unknown whether any unmeasured behavior was affected by the response‐independent stimulus delivery. Nevertheless, the results are consistent with other research reviewed in this paper indicating that adventitious reinforcement of either the target or alternative responses is not a likely outcome of NCR arrangements.

The available evidence thus suggests that explanations of the suppressive effects of NCR in terms of specific operant processes (satiation, extinction, and adventitious reinforcement) are incomplete. Operant satiation and extinction do not provide an adequate explanation of behavior reduction when implementing functionally irrelevant NCR. Further, if the outcomes of NCR are to be primarily explained in terms of its potential operant functions, it could be argued that adventitious operant reinforcement should be more common than the available evidence shows. However, as noted above, the evidence in favor of adventitious reinforcement resulting from time‐based schedules is scant.

In their discussion of the NCR literature, Phillips et al. (2017) noted that many of the potential disadvantages of NCR have not been borne out in practice and research:

For example, adventitious reinforcement of problem behavior is possible if scheduled reinforcer deliveries happen to occur shortly after problem behavior. Nevertheless, researchers have rarely reported such adventitious reinforcement effects, and when they have, those effects reversed quickly after they imposed an omission contingency wherein the delivery of the scheduled reinforcers was suspended if problem behavior occurred during the preceding few seconds. (Phillips et al., 2017, p. 358)

Thus, although adventitious reinforcement of either target responses or alternative responses seems possible in NCR arrangements (e.g., Ringdahl et al., 2001; Vollmer et al., 1997), it appears to be rare. Further, we are not aware of applied research demonstrating the development of stereotypic or superstitious behavior among individuals undergoing NCR as a treatment for problem behavior. Thus, the concerns expressed by Holden's (2005) colleague (and perhaps shared by other behavior analysts who were influenced by Skinner's work) have not been realized.

## Alternative Explanations of the Effects of NCR

### Contingency Strength and the Matching Law

Skinner's account of superstitious behavior was based on the notion that accidental response–reinforcer pairings increase *response strength*, defined as increased probability of a given response. Contiguity is important in Skinner's account; the superstitious outcomes will only occur if the reinforcer occurs close in time following a given response. This poses conceptual problems, given that operant reinforcers are otherwise defined in terms of response–reinforcer contingencies: The reinforcers must be more probable following a response than at other times. Indeed, *neutral contingencies*, in which reinforcers are delivered contiguous with target behavior but are equally probably at other times, can suppress target behavior (Borrero et al., 2002; Hammond, 1980). These findings show that response–reinforcer contiguity is not sufficient to account for maintenance of operant behavior; a positive contingency must be in place.

Therefore, some researchers have suggested that NCR may work to reduce the target behavior because of the availability of alternative sources of reinforcement (Fisher et al., 1999; Hagopian et al., 2000; Madden & Perone, 2003; Perez‐Gonzales, 2005). Consistent with the matching law (Herrnstein, 1970), this view assumes that under NCR schedules, a higher proportion of available reinforcement will be associated with behavior other than the problem behavior. The participant will match their response allocation accordingly; less time will be spent engaged in the problem behavior and more time will be spent engaged in other behaviors, which may partially consist of behavior related to seeking out and consuming the freely available reinforcers. Fisher et al. (1999) found that under NCR schedules, problem behavior was more likely to occur during interreinforcement intervals (i.e., when the reinforcers were not available), but it was unlikely to occur during reinforcement intervals (i.e., when the reinforcers were available).

Relative time allocation to problem behavior versus alternative behavior will likely be a function of the relative rates of problem behavior on the one hand and reinforcer delivery on the other. Lower rates of problem behavior and higher rates of reinforcer delivery will therefore likely lead to relatively higher rates of alternative behavior. It follows that thinning the schedule of NCR may lead to resurgence of problem behavior (Slocum et al., 2018; Vollmer et al., 1997). The extent to which *contingency strength* (i.e., the probability that reinforcers occur contingent on specific responses) predicts these outcomes could be explored in future research. This would require more extensive and systematic measurement of other behavior than has been undertaken in applied research to date (see section on applied–basic research synthesis below). However, we maintain that the current evidence suggests that antecedent functions of the stimuli delivered in NCR schedules likely play an important role. One way to conceptualize these antecedent influences is through Baum's (2012) concept of Phylogenetically Important Events (PIEs), which is also broadly consistent with the matching law.

### Phylogenetically Important Events

Baum's account (recently referred to as the *multiscale* account) posits that stimuli identified as reinforcing have the effect of inducing behavior that is directly or indirectly associated with survival (Baum, 2012; Segal 1972; Timberlake, 2004). For example, food induces behavior related to obtaining and consuming food (e.g., pecking by pigeons) and painful stimuli induce behavior relevant to escaping from or avoiding injury or death (e.g., fighting, fleeing). Baum (2012; 2018; 2020) referred to these stimuli as *Phylogenetically Important Events* (PIEs). According to this view, the effects of PIEs have been selected during the evolution of the species. Stimuli that are correlated with PIEs come to function as *conditional inducers* (Baum, 2012). Thus, obtaining food comes to evoke or elicit feeding‐related behavior (foraging, consuming, storing), and stimuli that are associated with pain or danger come to evoke or elicit any responses that function to escape or avoid the threatening situations. Inducers include stimuli that have been traditionally viewed as discriminative stimuli and eliciting stimuli, the primary difference between the two being the quantitative properties of the relation between the inducer and the response (i.e., latency and probability). Eliciting and discriminative stimuli are not viewed as qualitatively different, but rather as different points on a continuum. PIEs, therefore, not only serve to select behavior when they occur as consequences during the ontogenetic history of the individual, but also induce behavior that has been selected during the phylogenetic history of the species, as well as learned behavior that entered into previous contingencies involving PIEs (Killeen & Jacobs, 2017). An important contribution of the multiscale view is that it accounts for behavior that is induced by antecedent events in the absence of an operant contingency, while not being clearly accounted for by the respondent conditioning paradigm.

The view of reinforcing stimuli as inducers is attractive because it appears to account for a wide range of phenomena. This includes behavioral outcomes that have caused some difficulty for behavior‐analytic researchers in the past, such as avoidance. The fact that no measurable or observable events appear to reinforce avoidance responses is puzzling, but the view that aversive events (and signals associated with these events) induce behavior that functions to reduce the overall rate of the aversive events appears consistent with a wide range of studies on avoidance conditioning (Baum, 2020). This view is also broadly consistent with the numerous studies that have demonstrated that operant reinforcers appear to have effects that are similar to the effect of discriminative stimuli (Ingvarsson & Kahng, 2006), as well as the literature on autoshaping mentioned above (Brown & Jenkins, 1968; Williams & Williams, 1969). For the present purposes, recent basic research with pigeons and rats suggests that the concept of PIEs as inducers might provide an alternative account of some of the research findings on the effects of response‐independent schedules that seem inconsistent with the traditional view of operant reinforcement (Baum 2021; Baum & Aparicio, 2020; Baum & Grace, 2020).

### Antecedent Functions of Noncontingent Stimuli

The findings of Baum and colleagues suggest that noncontingently delivered stimuli might induce activity that contributes to the observed effects of NCR (Baum & Aparicio, 2020; Baum & Grace, 2020). Whether the induced activity consists of the target behavior or alternative behavior likely depends on the nature of the events as well as the state of the organism (Killeen & Jacobs, 2017). In some of the applied studies on functionally irrelevant NCR, the alternative stimuli have consisted of food, whereas the functional reinforcer might be qualitatively different, such as escape from demands (e.g., Lomas et al., 2010; Newman et al., 2021). In this account, food is a PIE that is likely to induce a variety of activities that involve obtaining, consuming, and maintaining access to the food. Ingvarsson et al. (2009) found that noncontingent delivery of food at the beginning of instructional trials increased compliance with tasks, even when problem behavior still resulted in removal of these same tasks. It is possible that food induces the behaviors we call compliance or cooperation because of a history of correlation between these behaviors and the availability of food. In other situations, NCR may induce target behavior, as when social stimuli have been shown to evoke or elicit vocalizations by infants (Bloom & Esposito, 1975). Social stimulation is likely a PIE for infants, or at least a conditional inducer due to its correlation with feeding and the warmth of physical touch. It seems plausible that it induces general activity for infants, including vocalizations that are then selected by operant contingencies. Similarly, it is likely that reinforcers that involve human interaction (i.e., attention) induce a variety of related activities that compete with problem behavior. Thus, NCR might engender collateral behavior that occurs along with behavior that has been reinforced in the past (Iversen, 1976). For instance, if compliance has been reinforced through the contingent delivery of attention, collateral behavior might include various social behaviors directed toward the therapist (e.g., orienting toward the therapist, talking to and touching the therapist). When attention is delivered noncontingently, these collateral responses may be evoked, shifting time allocation away from problem behavior. The extent to which this occurs likely depends on the history of the organism, which may explain the individual differences observed in research on functionally irrelevant NCR (e.g., Ingvarsson et al., 2009; Newman et al., 2021).

Lloveras et al. (2022) recently reviewed decades‐old research on self‐injurious and aggressive biting and reached similar conclusions regarding the potential antecedent functions of certain stimuli. As discussed by Hutchinson (1977) in a chapter in the *Handbook of Operant Behavior*, there is strong evidence that aversive stimuli (e.g., loud noises and other painful stimuli) and removal of reinforcers can elicit biting by humans and other organisms independent of an operant reinforcement contingency. There appears to be little doubt that humans and other species are evolutionarily prepared to bite in reaction to certain stimuli. These stimuli could then be conceptualized as PIEs in the multiscale account. Consequently, biting could be elicited by previously neutral stimuli that have been correlated with aversive events. These stimuli would be conceptualized as conditioned stimuli in a respondent conditioning account and conditional inducers in the multiscale account.

#### Do NCR Schedules Induce Problem Behavior?

Critics might point out that if NCR induces alternative behavior, function‐based NCR should also induce problem behavior (at least temporarily) because it involves time‐based delivery of stimuli that have been shown to maintain problem behavior in the past. However, given that NCR generally works to suppress problem behavior, this does not seem to be a common occurrence. One possible reason for the suppressive effects of NCR is that time‐based delivery of reinforcers might induce milder, less effortful forms of problem behavior that belong to the same response class as the more severe problem behavior for which an operant reinforcement contingency was arranged prior to the NCR intervention. A wealth of research has shown that milder and more severe forms of problem behavior tend to co‐occur, and unless restricted by a reinforcement contingency, milder or less effortful forms are likely to predominate (Borrero & Borrero, 2008; Magee & Ellis, 2000; Smith & Churchill, 2002; Warner et al., 2020). Because precursors and milder forms of problem behavior are not always measured in the literature on function‐based treatment of problem behavior, these effects may go unnoticed. Further, different types of antecedent effects might interact to prevent more effortful or severe problem behavior from occurring under these conditions. Specifically, time‐based stimulus deliveries might occur when the establishing operation for problem behavior is not in place (e.g., aversive demands discontinued, attention available), thus preventing problem behavior from escalating to more severe forms. It seems plausible that both operant and nonoperant processes interact to produce the outcomes typical of function‐based NCR schedules.

### Response‐Independent Schedules and NCR Summarized

In summary, the available evidence from applied research does not support the notion that adventitious reinforcement occurs because of response‐independent schedules. When NCR arrangements are used as behavior‐reduction procedures in applied contexts, there is little evidence of adventitious reinforcement of target behavior or other (alternative) behavior (e.g., Ingvarsson et al., 2008; Rey et al., 2020a; 2020b; Virues‐Ortega et al., 2013). On the rare occasions when the data suggest adventitious reinforcement of either the target behavior or alternative behavior, the effects appear to be short‐lived (e.g., Rey et al., 2020a; 2020b). It should be reiterated that no applied research to date seems to have included measurement of other behavior during NCR schedules beyond a narrow range of predetermined topographies. Further, suppression of behavior seems just as likely when function‐irrelevant reinforcers are delivered and functionally relevant reinforcers are still delivered contingent on the target behavior (Lomas et al., 2010; Phillips et al., 2017). This suggests that other operant principles, specifically extinction and abolishing operations (e.g., satiation), may not be sufficient to explain the behavior reductions that result from NCR procedures. Therefore, it might be time for applied behavior analysts and other behavioral scientists to take a more serious look at alternative explanations for the behavior changes that occur during NCR, explanations that might supersede the operant framework.

## Response‐Independent Schedules and NCR: A Basic–Applied Research Synthesis

The behavior of pigeons and other laboratory animals under response‐independent schedules does not appear to support an adventitious reinforcement/superstition explanation of the behaviors observed. Likewise, the reductive effects of undesired behaviors under response‐independent schedules (i.e., NCR) in applied settings do not appear to be under the control of adventitious reinforcement. Although the results observed in the laboratory are more easily explained as schedule‐induced species‐typical foraging behavior (e.g., Fernandez & Timberlake, 2020; Timberlake & Lucas, 1985), more research is needed to clarify the effects observed in applied settings with human clients. Below, we offer several suggestions that may aid in the study of response‐independent schedules in applied settings with the intent of better understanding behavioral changes that occur and what mechanisms may or may not be responsible for such change.

### Translational Analyses of NCR

To better understand the effects of NCR, a translational approach to the topic, meshing both an experimental and applied focus, seems necessary. In addition, it would be beneficial to borrow from other fields and areas of research, including behavioral ecology and ethology (Hackenberg, 1998). For instance, acknowledging and adjusting the relevant properties of antecedent stimuli necessary to make operants work (i.e., tuning) can facilitate our understanding of the importance of the organism in the learning experience (Fernandez & Timberlake, 2008; Timberlake, 2004). We consider three possibilities for such translational NCR studies: (a) measurement of other behavior, (b) contiguity–contingency transitions, and (c) application of omission contingencies.

#### Measurement of Other Behavior

As noted previously, early studies of response‐independent schedules often focused on one response, key‐pecking, and failed to maintain key‐pecking when transitioning from response‐dependent (fixed‐ or variable‐interval) schedules to response‐independent schedules of similar time lengths (e.g., Appel & Hiss, 1962; Lachter et al., 1971; Zeiler, 1968). Many of these researchers attributed the decreases in responding to other behavior being adventitiously reinforced. However, these other responses were not directly measured, and it was later research that, while systematically measuring multiple responses, observed a lack of idiosyncratic responding that questioned the validity of an adventitious reinforcement explanation of superstitious behavior (Staddon & Simmelhag, 1971; Timberlake & Lucas, 1985).

To better understand the mechanisms involved in the behavioral changes observed under NCR, applied researchers implementing NCR schedules would benefit from measuring all the behaviors that occur (Virues‐Ortega et al., 2013). This would include not just the undesired responses that decrease, but also the presumed increases in behaviors that replace the undesired responses. The use of behavioral inventories, better known as “ethograms” in the animal behavior literature (Altmann, 1974; Brereton et al., 2022; Lehner, 1998) could allow such measurement. The extent to which these behaviors are or are not idiosyncratic could improve our understanding of their potential function.^3^

#### Contiguity–Contingency Transitions

To the extent that some type of responding does increase because of the contiguous deliveries of NCR, researchers could examine whether reinforcing consequences could be made contingent on these same behaviors and produce similar results. As described earlier, previous research has found that functionally irrelevant stimuli may work equally well as functionally relevant stimuli during the delivery of NCR (e.g., Lomas et al., 2010). What is unclear is whether one could identify behaviors that do increase under NCR and produce similar increases in their occurrence when reinforcement is delivered contingently rather than contiguously. Certainly, the opposite has been demonstrated, where an undesired response such as head‐banging was shown to be sensitive to contingent reinforcement deliveries (Schaefer, 1970). However, to say that a behavior can be maintained by operant contingencies does not allow us to conclude that the same behavior is always the result of operant conditioning. If the delivery of NCR has important antecedent functions (e.g., as conditional inducers) that result in increases in behavior, we should be able to observe important differences when those stimuli are delivered as consequences contingent upon their occurrence. Relatedly, the literature on differential reinforcement of alternative behavior suggests that reinforcers that function to suppress behavior when delivered noncontingently will usually also increase alternative behavior when delivered contingently (e.g., Marcus & Vollmer, 1996).

#### Application of Omission Contingencies

The use of omission contingencies, where the occurrence of some response results in a delay of reinforcement, has played an important role in distinguishing between respondent and operant conditioning effects observed in the laboratory.^4^ For instance, the use of an omission contingency during an autoshaping procedure (Brown & Jenkins, 1968) allowed researchers to demonstrate that the key‐pecking responses observed were the result of stimulus–stimulus pairings of a lighted key with food (respondent conditioning), not the result of food following the key‐pecking (Williams & Williams, 1969). Similarly, if researchers examining the effects of NCR were to demonstrate that (a) other behaviors did increase, and (b) those behaviors could be maintained through the same stimuli delivered contingent on those responses, then the use of an omission contingency could allow one to discern whether the stimuli elicited or induced those responses, or whether the responses were emitted. Stated simply, if other behaviors that increased during NCR persisted when the occurrence of those responses delayed the continued delivery of the schedule, this would make a strong argument for antecedent (inducing or eliciting) effects of NCR.^5^

## Applied Implications

The preceding discussion should serve as a reminder of the multiple effects of any stimulus delivery. Thus, a general recommendation for behavior analytic practitioners is to consider potential antecedent effects of stimuli delivered as part of an NCR arrangement. In addition to the treatment of problem behavior, NCR schedules are often used in rapport building and as part of general behavior management procedures (universal protocols) to prevent problem behavior. Each of these uses of NCR will be considered briefly below.

### Implications for Problem Behavior Prevention

Research has repeatedly shown that humans tend to prefer contingent over noncontingent reinforcement schedules (Gover et al., 2022; Luczynski & Hanley, 2009). Presumably, this is because contingent schedules allow for reinforcement delivery when the relevant motivating operations are in place, therefore maximizing the value of the reinforcers. Nevertheless, due to the reliable behavior‐reduction effects of NCR schedules, NCR is often recommended as a part of universal strategies to prevent the occurrence of problem behavior in classrooms and other service settings (e.g., Williams et al., 2016). This may involve time‐based delivery of praise or attention, noncontingent access to toys, and so forth. We suggest that in using NCR schedules for these purposes, practitioners would be well advised to consider their potential antecedent functions. To the extent that the schedules might induce desirable behavior (e.g., appropriate social initiations, functional play), staff should be prepared to reinforce that behavior. However, the stimuli might also induce less desirable behavior due to conditioning history (e.g., if attention has predominantly been correlated with problem behavior in the past). It would be advisable to train staff to recognize these instances and adjust their approach accordingly.

### Implications for Rapport Building

Building rapport between interventionists and clients is an essential component of compassionate service delivery in applied behavior analysis (Taylor et al., 2019). Good rapport facilitates teaching interactions and reduces the probability of problem behavior (Kelly et al., 2015). Interestingly, procedures for establishing rapport involve an initial NCR phase followed by contingent reinforcement of social interactions and approach (McLaughlin & Carr, 2005). Based on the current discussion, it seems possible that NCR schedules induce activity such as orienting toward and approaching the interventionist that can then be selected by operant reinforcement. To the extent that this occurs, service providers would be well advised to systematically observe which responses are induced by specific stimuli, and program NCR schedules so that they include the stimuli that are most likely to induce behavior that can then be reinforced contingently.

### Implications for the Functional Assessment and Treatment of Problem Behavior

Lloveras et al. (2022) pointed out that aversive stimuli, such as the ones that are frequently presented in demand conditions of functional analyses, can elicit biting and other problem behavior independent of operant contingencies. As noted by the authors, this implies that it may not be possible to distinguish between problem behavior maintained by an operant escape contingency and behavior that is elicited (or induced) by antecedent aversive stimuli— both outcomes would look the same in multielement time‐series graphs. Functional analysis test conditions are similar to NCR schedules in that antecedent conditions involve time‐based stimulus presentation and removal (Hanley et al., 2003). Thus, it seems possible that test conditions for behavioral functions that involve removal of reinforcers (i.e., attention and tangible test conditions) could also lead to similarly indistinguishable outcomes. However, procedures to distinguish between these effects would likely involve repeated presentations of the aversive stimuli, which would most likely result in low social validity and acceptability of the assessment procedures and might lead to dangerous escalation of problem behavior. Practitioners should therefore be careful with employing such procedures, but translational research focusing on milder forms of behavior might help with understanding of these two kinds of effects, the relevant behavioral histories, and treatment implications.

B. F. Skinner identified the issue at hand as early as 1953, when he stated: “Aversive stimuli elicit reflexes and generate emotional predispositions which often interfere with the operant to be strengthened” (Skinner, 1953, p. 172). Because the purpose of a functional analysis is to identify an effective treatment as soon as possible, practitioners might be well‐advised to assume that any differentiated assessment outcomes could entail both types of effects: maintenance by operant contingencies and induced or elicited responding by antecedent stimuli. This would be consistent with recommendations for trauma‐informed care—indeed, some of the antecedent effects of the relevant stimuli might be due to a history of trauma (see Rajaraman et al., 2022, for a detailed discussion). Lloveras et al. (2022) astutely suggested demand fading as a potentially effective treatment for behavior induced by antecedent stimuli, because it involves both gradual exposure to the aversive stimuli and pairing with reinforcement. In general, treatment could start with noncontingent access to reinforcers that occasion behavior and emotional states that are incompatible with problem behavior escalation. Individualized measures of indices of happiness and relaxation could help in that regard (Metras & Jessel, 2021). This would set the stage for treatment involving gradual exposure to aversive stimuli and skill building focusing on functional communication. The resulting treatment process would allow the client greater control over their environment and potentially reduce the likelihood of the kinds of “elicited reflexes and emotional predispositions” that Skinner discussed.

## Conclusion

Our understanding of the effects of response‐independent schedules has been greatly influenced by the work of Skinner and other behavior analysts. As a result, it is to be expected that we continue to look for operant reinforcement effects in NCR, as well as all behavior. However, as our review illustrates, in both the basic and applied research, little support exists for an adventitious reinforcement effect observed under response‐independent schedules. The use of the concept of adventitious reinforcement and superstition may have been largely controlled by learning histories rather than empirical evidence.

Aside from the difficulty in defining both operant and respondent reinforcement in the absence of a contingency (Carr, 1996; Poling & Normand, 1999; Rescorla, 1967; Staddon, 1992; Vollmer, 1999), the concept of noncontingent reinforcement has, in the past, led some behavioral scientists toward attempting to understand the effects of NCR through an operant‐only viewpoint. However, there is also a history within behavior analysis for identifying the importance of PIEs, as those presumed to exist for processes such as induction (Baum, 2012; 2018). Likewise, Skinner spent a considerable amount of his later writings on the importance of both phylogeny and selection on behavior (Skinner, 1975; 1981; 1984; 1987; 1989). As he stated:

Natural selection prepares an organism only for a future that resembles the selecting past. This is a serious limitation, and to some extent it was corrected by the evolution of a process through which a different kind of consequence could select additional behavior during the lifetime of the individual. The process is called operant conditioning and the selecting consequence a reinforcer. (Skinner, 1989, pp. 114–115)

It is therefore critical that we, as behavior analytic scientists and practitioners, consider mechanisms other than operant conditioning when exploring the effects that occur during response‐independent schedules. This does not preclude that operant contingencies are involved during NCR. It is equally possible that different stimuli, schedules, and contexts have different purposes and, therefore, different explanatory variables. It simply provides an expanded realm of options for the discovery of the functions of the behaviors we observe, operant or otherwise.

## Footnotes

^1^
A positive contingency is one in which the probability of reinforcement is greater following a target response than at other times. By extension, greater probability of reinforcement at other times implies a negative contingency, whereas a neutral contingency involves equal probability of reinforcement following the target response and at other times (Borrero et al., 2002; Hammond, 1980).

^2^
At least one study has suggested that when fixed‐time (FT) schedules are implemented following a fixed‐ratio (FR) or fixed‐interval (FI) baseline, greater reduction in target behaviors may occur when the FT schedule is dissimilar from the baseline schedule (Ringdahl et al., 2001). This may suggest that adventitious response–reinforcer pairings are more likely when the transition from contingent to noncontingent reinforcement involves a similar schedule (e.g., FI‐30s to FT‐30s), but the evidence for this occurring was inconsistent across the three participants.

^3^
Applied and translational studies on “other” behavior in DRO schedules could also provide a model for this research (e.g., Hangen et al., 2020; Iannaccone et al., 2020; Jessel et al., 2015).

^4^
In the applied literature, this kind of contingency is typically referred to as a resetting interval DRO (differential reinforcement of other behavior; Gehrman et al., 2017).

^5^
Momentary DRO (mDRO) schedules, in which a reinforcer is delivered if the target behavior is not occurring at the precise moment a predetermined interval ends, offer a potentially similar type of control (e.g., Hammond et al., 2011). However, caution should be used in interpreting their effects because the absence of a consequence for some event (i.e., mDRO) is not the same as a direct punishing consequence for engaging in the operant response (i.e., omission contingency).
